# Social-ecological niche construction for sustainability: understanding destructive processes and exploring regenerative potentials

**DOI:** 10.1098/rstb.2022.0431

**Published:** 2024-01-01

**Authors:** Christian Dorninger, Lumila Paula Menéndez, Guido Caniglia

**Affiliations:** ^1^ Konrad Lorenz Institute for Evolution and Cognition Research, Martinstraße 12, Klosterneuburg 3400, Austria; ^2^ Institute of Social Ecology, University of Natural Resources and Life Sciences, Schottenfeldgasse 29, Vienna 1070, Austria; ^3^ Department of Anthropology of the Americas, University of Bonn, Oxfordstraße 15, 53111 Bonn, Germany; ^4^ Department of Evolutionary Biology, University of Vienna, Djerassiplatz 1, 1030 Vienna, Austria

**Keywords:** niche construction, Anthropocene, social ecology, sustainability

## Abstract

Through the exponential expansion of human activities, humanity has become the driving force of global environmental change. The consequent global sustainability crisis has been described as a result of a uniquely human form of adaptability and niche construction. In this paper, we introduce the concept of *social-ecological niche construction* focusing on biophysical interactions and outcomes. We use it to address destructive processes and to discuss potential regenerative ones as ways to overcome them. From a niche construction point of view, the increasing disconnections between human activities and environmental feedbacks appear as a success story in the history of human–nature coevolution because they enable humans to expand activities virtually without being limited by environmental constraints. However, it is still poorly understood how suppressed environmental feedbacks affect future generations and other species, or which lock-ins and self-destructive dynamics may unfold in the long-term. This is crucial as the observed escape from natural selection requires growing energy input and represents a temporal deferral rather than an actual liberation from material limitations. Relying on our proposal, we conclude that, instead of further taming nature, there is need to explore the potential of how to tame socio-metabolic growth and impact in niche construction processes.

This article is part of the theme issue ‘Evolution and sustainability: gathering the strands for an Anthropocene synthesis’.

## 1. Introduction

Human social and natural systems have coevolved over millennia as social systems shape the natural environment and the environment shapes the emergence and development of social systems. The recent decades, however, have brought about an unprecedented acceleration and intensity of this reciprocal interference with profound implications for the future stability of human–nature coevolution [1–3]. Through the exponential expansion of human activities, humanity has become the driving force of global environmental change [4–6] and influences a substantial and growing part of natural ecosystem trophic interactions and energy flows [7,8].

The consequent global sustainability crisis has been described as a result of a uniquely human form of flexibility and niche construction [9–11]. All species have ecological niches, and humans are no exception [12,13]. Like other organisms, humans adapt to and at the same time modify their environment in potential fitness-beneficial ways [14–16]. When such modifications entail evolutionary consequences and change the selective regime that organisms are exposed to, they are called niche construction [17]. Niche construction has consequences for the dispersal and reproduction resulting from causal feedbacks between what organisms do and constraining responses from the environment [18]. The human niche is distinct in the degree to which it involves the transmittance and inheritance of material culture as well as cognition, behaviour and cultural knowledge [14,15] that can drive evolutionary selection on human genes [16,19]. Through the evolution of new cultural traits, such as technologies and novel forms of social organization, humans construct niches that work to suspend and defer self-constraining feedbacks from the environment in an unprecedented manner [20–22]. These changes contributed to the construction and expansion of the human evolutionary niche.

Additionally, humans also modify their environments independently of adaptive needs or pressures [23]. Starting in the Anthropocene [4,24], human societies largely have managed to circumvent limitations and self-constraining feedbacks imposed by natural cycles and competitors [23,25,26]. However, focusing on the socio-cultural dimension of advanced human niche construction risks to neglect that humans still occupy, construct and possess ecological niches—together with all their material and biophysical interdependencies, limitations, environmental impact and evolutionary consequences [9,15]. Human societies are still confronted with natural limits, environmental feedbacks and biological competitors, both locally and globally, but in a different and less obvious way than in the past. Indeed, the supposed escape from natural selection requires growing energy input and represents a temporal deferral rather than an actual liberation from material limitations [27,28]. The concept of ‘planetary boundaries’ attempts to grasp and quantify such limits on a global scale, which, when transgressed, might lead to irreversible and potentially catastrophic paths for the future of humanity [29,30].

Whereas seminal contributions have translated biological and ecological niche construction principles into the social sciences [31,32], we still lack concrete conceptual frameworks and narratives to grasp complex social-ecological phenomena from a niche construction perspective, while also being sensitive to spatiotemporal differentiation, global interdependencies and biophysical implications. Even though particularly contributions on a theory of long-term anthropogenic ecological change (anthroecology theory) represent an important point of reference in this regard [33], relevant questions regarding the evolutionary implications of global heterogeneity between social-ecological niches, their interdependencies, and the distribution of natural resources—as well as our highly problematic ‘deep evolutionary dependence on external energy’ [34] and the ‘self-endangering evolutionary trap’ from counteractive niche construction [35,36]—remain insufficiently explored.

In order to highlight the main features of social-ecological processes of niche construction, in this article we focus on the biophysical realm of human niche construction by combining niche construction theory [18,31] with social-ecological systems science [37,38]. In doing so, we propose to extend the niche construction lens towards a systemic and comprehensive approach to the causal interrelationships between sociocultural and environmental co-evolutionary processes that have led to the current planetary crisis. Applying such an extended niche construction perspective, we draw attention to specific, formerly under- or unrecognized phenomena of cross-scale human-nature interactions and put them into a long-term evolutionary context, e.g. functional interdependencies in niche construction, inequality in resource access, and the simultaneousness of niche construction processes at different places.

A central goal of this paper is to further develop the argument that for the analysis of social-ecological niche construction processes, it is crucial to focus not only on certain technologies that facilitate human–nature interaction, but also on the material and energetic as well as on the social and cultural underpinnings of these technologies. Whereas they are key for how humans create their niches, technologies also have a strong redistributive character of environmental goods and burdens between geographically separate human niches, involve the large-scale extraction and movement of resources from the environment, and create pathway dependencies [39,40]. All of this becomes even more relevant for industrial technologies which rest on the availability of large amounts of fossil fuel energy, metals and construction minerals [41,42].

In the following §2, we conceptualize social-ecological niche construction by characterizing social-ecological causation, inheritance, and the biophysical dimensions of social-ecological niches. We then discuss the potentially destructive consequences of industrialized niches in §3, and possible ways for entering regenerative niche construction processes in §4, before we conclude in a final §5.

## 2. Conceptualizing social-ecological niche construction

In this section, in order to develop a concept of social-ecological niche construction, we theoretically integrate insights from cultural evolution [43] and from social-ecological systems science [37].

### (a) Social-ecological causation

**Figure 1.**
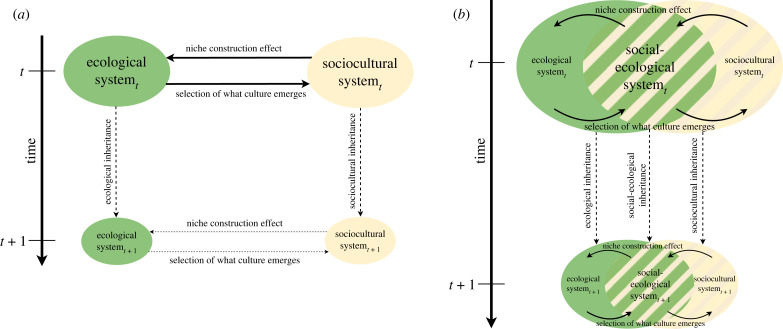
(*a*) The human niche construction framework. It shows the reciprocal interference between natural (ecological) and cultural (sociocultural) systems via niche construction and natural selection. Both systems involve an inheritance scheme over time. Adapted from Laland *et al*. [44,45]. (*b*) The social-ecological system exists as a hybrid at the overlap of the ecological and the sociocultural system. Social-ecological niche construction happens at the interface of ecological and cultural spheres. Social-ecological patterns are inherited over time. Note that distant future is indicated by smaller-sized system ovals.

Figure 1*a* visualizes human niche construction process following Odling-Smee *et al.* [43–45]. Even though the human niche is a complex interface intersected by social and ecological dimensions, here the two are separated for analytical purposes to distinguish ecological and sociocultural modes of inheritance (left-hand side of figure 1). While the environment (ecological system) exerts selective pressure on the human sociocultural systems, through concerted action, societies also modify their natural environment to decrease the effects of selective pressure and to make it more useful for their own purposes (which also entails unintended consequences of human activity). This is the basic human niche construction process. Its realm of causation captures both socio-cultural and material-ecological dimensions, and its evolutionary character leads to a sociocultural inheritance over time, similar to evolutionary inheritance within ecological systems.

Building on this representation, figure 1*b* introduces an overlapping sphere where the biophysical structures of human society (i.e. domesticated animals, human-made artefacts, humans) are treated as hybrids between the ecological and sociocultural sphere of causation, i.e. coupled social-ecological systems that are subject to both spheres of causation and hence to both types of inheritance. Thus, biophysical structures of society are also subject to ecological causation: they are either living entities (humans, domesticated animals)—which need to maintain their metabolism, grow, reproduce, and die; or they are human-made artefacts—which are often formed by inorganic and non-renewable materials (like buildings, streets, cars, books, etc.), but still made of natural resources that decay over time if not maintained properly. In the field of social ecology [46,47], the ‘biophysical structures of human society’ are generally understood as the human populations themselves, the human-made artefacts (from clothing to large-scale infrastructure, basically every physical entity shaped by humans with a certain purpose), and domesticated animals which are under human reproductive control. Delineating this hybrid sphere enables the analysis of coupled social-ecological causation and inheritance.

However, adapting this social-ecological system understanding for a niche construction perspective requires a partial extension and detailing: from a niche construction perspective also ‘domesticated’ plants are to be considered as part of this hybrid sphere. This is because they are specifically bred, reproduced, genetically modified, sown and harvested and have a significant impact on inheritance schemes (e.g. agricultural development or digestive systems). The detailing pertains to specific microbiomes such as the rhizosphere—the plant root and soil microorganisms' interface [48] or the human (and domesticated animal) gut microflora [49].

All these elements belonging to the hybrid social-ecological system sphere, are also subject to sociocultural causation. That is, humans, domesticated animals and plants live and reproduce according to evolved and inherited social codes and narratives that guide human–nature interactions for expanding and optimizing the procurement of resources and for fulfilling an individual's metabolic purposes [21,50,51]. For this, and in contrast to other species, modern human societies maintain—in addition to their basic biological metabolism for their own individual bodies—an extended social metabolism that vastly exceeds their basic metabolism in terms of matter and energy throughput [21,34,50]. The social metabolism includes all resources extracted from the environment that are needed to grow and maintain human-made artefacts like buildings, vehicles, machines, among others. The evolution of this extended social metabolism was key for human societies to construct niches on larger scales and at multiple levels. It allows reproducing not only the human population as such but additional social-ecological structures, to construct and maintain artefacts and infrastructure, as well as to continue the process of domestication of animals and plants.

### (b) Social-ecological inheritance

Elaborating on the overlapping hybrid sphere in figure 1*b* in terms of inheritance, we suggest that this sphere entails a distinct form of *social-ecological inheritance* which is more than and different from what has been described either as ecological or as sociocultural inheritance [22,52]. That is, analysing how humans and human societies evolve and reproduce requires an interdisciplinary perspective integrating ecological and sociocultural inheritance insights into a social-ecological mode of inheritance. The traits that are inherited and spread here are intrinsically relational and pertain to how humans and human societies interact with their local or distant environment and with one another over time.

To start with a prominent example in cultural evolution studies, the evolution of agricultural practices requires an analysis of the natural and sociocultural traits which are inherited to form new traits, including but not limited to, types of human–nature interaction (how human invest energy to extract resources), social forms of organization (how humans are dividing tasks), changes in human diet across time, human mobility patterns, environmental changes and the process of selecting organisms, breeding and modifying them for specific purposes [53]. Since the Neolithic Revolution, humans colonized nature to transform natural systems for higher usability, i.e. through agricultural practices, species breeding, animal domestication, and the development of specific technologies. The maintenance of such colonizing efforts also had profound effects on societal structures and institutions [46], e.g. settling down, dividing work tasks, constructing specific artefacts, and thereby building a social-ecological legacy and inheritance, e.g. inheritance schemes of human–nature interaction patterns, material inheritance [33], institutions, division of labour, norms and values, which are transmitted among human groups and through generations over time.

More generally, social-ecological inheritance pertains to how patterns of human–nature interaction are transmitted and adopted while also changing over time. It involves how societies reorganize themselves to fit the reorganized environment (the constructed niche). This kind of inheritance also impacted on the evolution of, e.g. the human digestive system in such a way that societies in which dairying products became relevant, humans developed the persistence of the enzyme lactase in adulthood, which is usually shut down after postweaning [49]. Another specificity of social-ecological inheritance is that keeping up the states and functions of social-ecological systems structures requires continuous efforts (labour, energy, coordination, agreement) from preventing the ecological system to exert limiting feedback again and from ‘re-naturalizing’ social-ecological system properties. Continuous monitoring and re-adjustment are required to maintain the desired state of the environment [46,50] and, thereby, form strong patterns of social-ecological inheritance. Legacy from infrastructure and technologies is yet another important part of the social-ecological inheritance scheme, for it relates to forms of social organization that allow for coping with the legacies of physical infrastructure maintenance technologies, e.g. nuclear waste treatment, control of geo-engineering efforts, carbon capture and storage, among others.

In sum, progressive processes of social-ecological niche construction act to decrease direct exposure to the environment [10,54,55] and it has intensified significantly with the industrialization of agriculture [56], the emergence of global trade regimes [57–59], and the associated division of labour in societies. However, also in the Anthropocene natural forces are not completely under human control but are only temporarily suppressed and resurface occasionally with often dramatic consequences, as the COVID-19 pandemic illustrated [60]. Importantly, this ‘resurfacing’ of constraining environmental feedback—due to, e.g. biodiversity loss, environmental pollution, global climate change, emerging diseases—is predicted to happen more often and more regularly in the future [61,62].

### (c) Biophysical dimensions, from local to global

Especially since the Industrial Revolution, the metabolism of societies goes beyond interactions with the biosphere only, but includes the massive extraction and use of inorganic materials, especially of fossils fuels like coal, oil, and natural gas from the lithosphere [34]. In coordinated action, humans facilitate large-scale social metabolic processes of resource extraction and stockpiling of built-up infrastructure and artefacts [41]. With this increasing social metabolism (higher input of natural resources, larger socioeconomic stocks, higher outputs as emissions and wastes generated) humans are driving more and more changes at the global environmental sphere, e.g. climate change, soil degradation, environmental pollution. The throughput and stockpiling of biophysical resources are used to construct niche structures via technologies that help dampening the constraining effects of the natural environment on societies, at least in a spatially and temporally limited manner.

Accessing the lithosphere for non-renewable resources is no unique feature of industrialized societies. Millenia ago humans already extracted materials from the lithosphere because of the increased need for metals and other non-metallic minerals: compare the Stone, Bronze and Iron Ages all around the world. However, the scale of resource extraction makes the difference [63,64]. Recent work demonstrates that human-made material outputs (i.e. the mass of all global buildings, infrastructure or plastic, etc.) now outweighs all global living biomass (i.e. plants and animals living on the planet; human bodies only make up about 0.01% of global biomass) [65].

Industrial societies extract and process vast amounts of construction minerals and metals to build up industrial complexes, transportation systems, machines and infrastructure [41,66]. Moreover, the ‘game changer’ which lifted human activity to another level [2] was clearly the massive use of fossil fuels for energy generation. Fossil fuels are transformed to boost and substitute natural productivity and biomass use (free human activity from land-based environmental constraints) [21,64], substitute animal and human labour power [28], and enable long-distance trading [67]. Still, (i) fossil fuel resources are highly unequally distributed in the world system and (ii) their use comes with well-known long-term legacies like anthropogenic global climate change, resource dependency, pollution and depletion. It is important to recognize that not all social-ecological niches existing today are constructed and maintained with the same level of technology and resource use (some do not have access to fossil fuels at all), but they are connected and partly interdependent in their existence; compare studies on ecologically unequal exchange and the unproportional access to resources and sinks [28,68].

**Figure 2.**
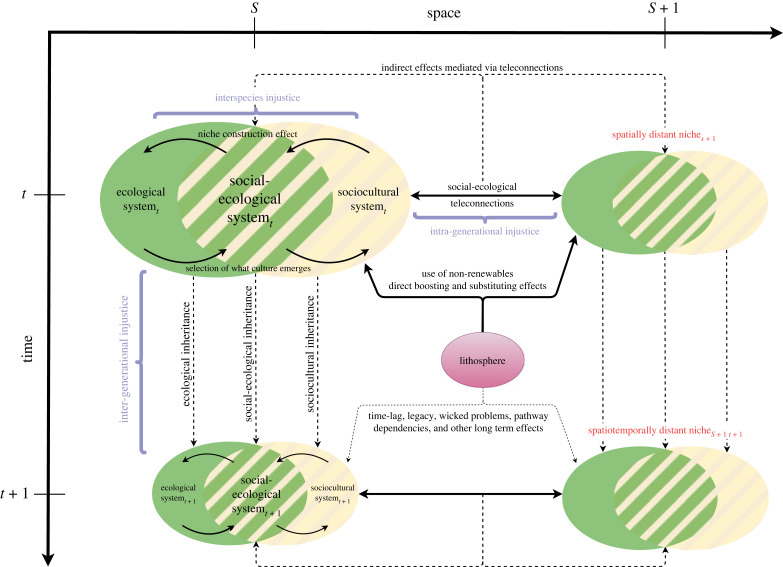
The extended industrial social-ecological niche construction framework. The temporal dimension is extended by a spatial dimension to capture interdependencies with spatially distant niches. The effects of accessing the lithosphere for non-renewables are indicated for all niches, however they differ for current and future generations. Curly brackets in blue indicate potential sustainability issues emerging from industrial niche construction: indicated as potential injustices within current generations (intra-generational injustice), between current and future generations (inter-generational justice), as well as human and non-human species (interspecies justice). Note that spatial and temporal distance is indicated by smaller-sized system ovals.

These aspects of modern social-ecological niche construction are crucial. The modern, wealthy and industrialized societies significantly and increasingly disconnect their development from their surrounding natural biospheric productivity, such as by artificially boosting and substituting natural biospheric productivity with non-renewable inputs and by outsourcing production steps through importing biophysical goods from distant places—as indicated in figure 2 [28,69]. The increasing biophysical disconnect between humans and their surrounding natural environment is a prime example of successfully circumventing limitations and self-constraining feedbacks of natural cycles, which is a crucial feature of counteractive niche construction, i.e. one that counteracts self-limitation [15,18,70]. However, disconnecting human activities from regional natural productivity does not completely escape but merely temporarily avoids and defers direct consequences of limitation and self-constraining feedbacks from the environment as the disconnection requires continuous external resource inputs. There are three main practical ways for this deferral: (i) local environmental constraints can be circumnavigated via access to distant biotic resources (biomass) which supplement local provision, (ii) local natural productivity can be boosted and substituted by relying on non-renewables for agrochemicals and an otherwise industrialized agricultural system, and (iii) generated (hazardous) waste and waste-intensive production or extraction (like mining) can be transferred to spatially distant niches [28,71–73]. All three ways are feasible options to escape local source and sink constraints, however, they run the risk to violate intra- and inter-generational justice concerns for depleting resources elsewhere and causing pollution as well as long-term changes to the global environment.

Consequently, for a comprehensive analysis of modern human niche construction, it is not sufficient to focus on the apparent on-site interaction with the biosphere, but the scope has to be widened to encompass the exploitation of non-renewables from outside the biosphere as well as the large-scale appropriation of resources from and shifting of wastes to spatially distant places. Figure 2 conceptually depicts the modern social-ecological niche construction process that explicitly acknowledges (i) the effects occurring from the industrialization of land-use and other sectors of the economy (through accessing the lithosphere for resource extraction of non-renewables like fossil fuels and metals); and (ii) the emergence of large-distance trade regimes and the associated effects occurring over distance, like the outsourcing of resource extraction and waste disposal, i.e. social-ecological teleconnections [28,74].

To facilitate future empirical research from the reflections above, we tentatively suggest some methods that may be useful to analyse social-ecological niche construction processes (figure 2). Concrete empirical investigations will depend on the specific research question, foci and scales:

1. The system state of the social-ecological system in focus can be assessed using material and energy flow accounting methodology [75] by collecting quantitative data on the biophysical structures of the system (human population, domesticated animals and plants, human-made artefacts), including the so-called socioeconomic stocks [66].
2. The state of the ecological system (natural productivity, biodiversity, ecosystem functions) and the degree of human domination can be approximated via the human appropriation of net primary production (HANPP) indicator [7,76].
3. Accounting for resource extraction from ecosystems (biomass) as well as from the lithosphere (all other non-renewable materials) is well documented by social metabolism studies [51].
4. Social-ecological teleconnections—i.e. interconnections between spatially separated places—are best approximated via the assessment of direct and indirect trade linkages, including spillovers and the distribution of environmental goods (resources) and burdens (e.g. outsourced land-use change, emissions, biodiversity loss, pollution, etc.). Environmentally extended multi-regional input-output analysis is a prominent and regularly used methodological approach to measure such teleconnections [77,78].
5. Inheritance structures can be elucidated by historical analyses and by a focused analysis of the stocks and flows of the current niche that are decisive for societal inheritance (i.e. institutions, operations and paradigms, ecological patterns in fauna and flora, as well as biophysical structures including stocks, material culture and patterns of human–nature interaction).
6. In addition, qualitative data appear useful to approximate the sociocultural codes, cosmovision, policies, paradigms and narratives, that guide the niche construction effect [79].

## 3. Destructive features of industrial niche construction

As discussed in the previous section, consequences of niche construction are usually causal feedbacks between what organisms do and responses from the environment. In industrialized societies, niche construction intensified but simultaneously brought about a spatiotemporal biophysical disconnect between the engineering humans and the responding environment, indicating that these causal feedback loops appear broken to the humans involved. The combination of the obscuration (out of sight either because impacts are happening elsewhere or in the distant future) and concurrent increase of human impacts leads to deleterious self-reinforcing feedbacks as it only defers environmental consequences or outsources them, which might even exacerbate negative impacts on the environment in total, but, crucially, does not resolve them. What is arguably one of the main success features of human evolution—the development of cultural tools and programmes that anticipate and avert negative feedbacks from the environment—might, in an industrialized and globalized world of the Anthropocene, endanger humanity's own survival and lead to violent conflicts or collapse in the future [80,81].

While humans are exceptionally well adapted to counteract environmental change, niche construction typically benefits the constructor in the short term, but need not benefit other species that share its ecosystem or future generations [44]. The use of non-renewables from outside the biosphere has a significant evolutionary influence on how social-ecological niches are constructed. While the immediate local niche construction effect—in terms of making the natural environment more useful for humans and circumventing limiting feedbacks—will be increased, the large-scale exploitation and use of fossil fuels, metals and other minerals creates adverse long-term legacy effects. As for example, risk spirals of further engineering and controlling natural systems (e.g. climate engineering, deep sea mining, nuclear power), the effects of climate change, irreversible biodiversity loss, or environmental pollution impede the option space of future generations. Industrial niche construction is, therefore, based ‘on credit’, i.e. it exceeds ecosystems’ regenerative source and sink capacities and transgresses planetary boundaries delimiting the safe operating space of humanity on Earth.

Making these dimensions more explicit, figure 2 indicates where potential sustainability issues of social-ecological niche construction might arise (in blue)

1. … through niche construction processes humans appropriate or destroy an unproportionally large share of the trophic energy from ecosystems over other species [7] which threatens biodiversity conservation and *interspecies injustice* [82];
2. … in unbalanced trade relationships with spatially distant systems that fuel uneven conditions for niche construction by unequal access to resources and shifting of environmental burdens, which hampers *intra-generational injustice* [68,83];
3. … in the large-scale reliance on non-renewables which decreases *inter-generational injustice* for irreversible destruction of healthy ecosystems, changing climatic conditions and the exhaustion of non-renewable materials [84].

These issues are especially important because effects of niche construction can also involve destructive consequences which involve lasting changes to the physical environment and modifies selection not only for humans but also for other species [9,70]. In fact, the global expansion of counteractive social-ecological niche construction, e.g. through the expansion of industrial agriculture, industrial infrastructure and urban development, causes increasing habitat degradation, global climate change, pollution, deforestation, biodiversity loss and often destroys ecosystems' integrity [44]. But the immediate effects and impact on natural systems are often out of sight because environmental burdens are increasingly shifted to spatiotemporally distant niches [85,86]. A theory of social-ecological niche construction must, therefore, more explicitly aim to capture issues of *inter- and intra-generational injustice*, like inequalities in distribution of these environmental goods and burdens, unequal power relationships, and interdependencies in niche construction processes to better understand how local niches function differently to form global human evolutionary pathways.

While culture enhances the effect of human niche construction to defer natural limitations, it also increases risk spirals and wicked legacies associated with this process [87,88]. That is, while trying to transform danger and reduce primary uncertainties, humans produce new, secondary risks, as for example the large-scale use of fossil fuels to reduce uncertainties relating to energy provision led to global climate change which, in turn, shall be countered by geo-engineering measures with unforeseeable consequences for global natural cycles. In a similar vein, wicked legacy relates to legacy from human-made artefacts and biophysical structures that once constructed and set in place, require continuous monitoring and social dedication to keep them functionable and not dangerous, e.g. nuclear power plants, large dams, space debris, etc. Thus, the unintended side-effects that come with the domination of nature are increasingly costly and risky for society; compare the so-called ‘Anthropocene risks’—cross-scale systemic environmental risks with global effects [62]—and the super wicked problems [89,90] that attempt to capture urgent, hard-to-solve problems with far-reaching consequences but lacking authority to tackle them. Eventually, the continuous suspension of self-limiting feedbacks and increasing environmental impact might add up to a situation where societal fitness—after steadily increasing—suddenly drops significantly; compare the ‘cliff-edge model’ [91], which resembles warnings expressed in the ‘planetary boundaries' concept [29,30].

That is why in the era of *Anthropocene-risk* [62], cultural evolution has to work faster than natural selection to prevent collapse [20]. Yuval Noah Harari [92] frames this dilemma as a double race which humankind is locked into: on the one hand we feel compelled to speed up the pace of scientific progress and economic growth (i.e. avoid the action of natural selection in the short-term and close range), on the other hand, we must stay at least one step ahead of ‘ecological Armageddon’ (i.e. continue increasing long-term destructive niche construction). Therefore, constraining feedbacks of already existing stocks on societal functioning need to be considered more explicitly for inherent path dependencies and lock-ins [88]. In the future, constraining the feedback from human-made environments (like power plants, infrastructure, waste disposals etc.) may be more decisive for path-setting than constraining the feedback from the natural environment (e.g. climatic conditions, natural productivity).

## 4. In search of regenerative niche construction processes

Clearly, no niche construction process can be classified as exclusively destructive or regenerative, because there will always be winners and losers, i.e. species that thrive and reproduce in the course of presumably destructive industrial niche construction [93] or the global spread of domesticated animals and plants, while other wild animals and plants are decimated. However, applying methods from the list in §2c and other scientific approaches allow us to operationalize niche construction effects into measurable pressure and impact indicators which can be used to—at least on aggregate—identify, monitor and distinguish destructive and regenerative patterns of niche construction. In addition to apparent on-site impacts, it is crucial to also comprehensively assess indirect, remote and long-term effects of human niche constructing activities. Such activities can be identified as destructive when they are based on ecological ‘credit’ for using more resources than natural ecosystems can provide and regenerate, because of associated immediate negative environmental impact they cause—compare research on the sixth mass extinction [94,95]—and because of its self-reinforcing nature involving adverse long-term legacies and risk-spirals. By contrast, regenerative niche construction processes also do not re-establish some sort of ‘pristine nature’ but they can be understood as processes that enable a thriving human society while not transgressing planetary boundaries (e.g. concerning climate change or biodiversity loss) or disproportionally shifting environmental burdens to spatiotemporal distant human generations via destructed aquatic and terrestrial ecosystems or degraded soil that cannot sustain life of current populations into the distant future.

A central aspect of the difference of classifying niche construction processes as destructive or regenerative boils down to the question of whether or not it may be possible to achieve a permanent decoupling of (growing) human activities and environmental impact on the global scale. Current empirical research and meta studies indicate that this is not (yet) the case [93,94,96]. Hence, acknowledging that the observable industrial and globalized counteractive niche construction processes do not lead to regenerative but rather destructive environmental outcomes in the long run—and therefore involve self-destructive features for future generations—raises the need to look for alternatives. The main questions thus revolve around whether, to what extent, and how social-ecological niche construction processes can be steered, triggered, irritated or navigated with the awareness of the uncontrollable and unpredictable nature of these evolutionary processes. While humans have evidently shaped the course of evolution over millennia, it is far less clear how evolutionary causation on larger scales and over long periods of time can be deliberately influenced towards more regenerative processes that allow for staying within planetary boundaries while meeting intraspecies, inter- and intragenerational justice. Hence, there is need to better understand how unsustainable and destructive niche construction processes may potentially be tamed. This would imply establishing processes that go beyond established ways of merely avoiding self-limiting environmental feedbacks in the short term and only for the constructing agents [81,95].

Another key question for reflecting on future niche construction is, thus, how to break out of the vicious cycle that pulls global human society further and further into risk spirals (how to regenerate)? The continuing large-scale transformation of ecosystems and changing of the global climatic conditions evoke new forms of social responses, which are again often associated with ecomodernist approaches of controlling and engineering nature: genetic engineering, ecosystem engineering, or geoengineering [1,6]. Researchers describe two main alternative pathways as either (i) increasing geoengineering and counteractive niche construction to further delay negative feedbacks while enhancing positive feedbacks [97] of growing colonization and metabolism, or (ii) taming instead human growth and impact, which would require a focus on and support of regenerative niche construction processes [98–100].

Following this latter option (ii), future-oriented regenerative processes would need to consider the different scales of complex social-ecological systems, from micro to meso and macro. Among many, we may be able to learn what it means and what it implies to initiate and maintain regenerative niche construction processes from studies addressing diverse biological systems, from cells and tissues to entire ecosystems [101,102]. Existing studies have generated a great amount of knowledge about multiple aspects of regeneration within living systems and they are pushing the boundaries of our knowledge about the logic that governs regenerative processes. If we can abstract the logic of regeneration in one system and compare how it operates within each other type of system, we should be able to gain even more insights into what we are missing from our understanding of regenerative processes more generally, from cells to ecosystems and social-ecological systems.

Further, it will be important to be able to learn from smaller scale interventions and processes of experimentation. In this way, it will be possible to find out more about alternatives and regenerative processes to make use of natural resources and creating transformative human–nature relationships. Examples may be the numerous real-world experiments that show how regenerative rather than destructive interconnections and social-ecological configurations may be created on small scales and how their interconnections and co-evolution might lead to regenerative global dynamics [103]. So-called ‘Seeds of a Good’ Anthropocene present radically different forms of living involving fundamental change in human–environment relationships, including changes in values, cultures, worldviews, and even the power and gender relations influencing social norms and behaviour [104]. The tensions and opportunities that emerge from such examples may inform and inspire the patchwork emergence of regenerative pathways in multiple regions and through a wide diversity of pathways.

Similarly, it will be essential to learn from newly evolving research on ‘collectively defined self-limitation’ [105], ‘sufficiency’ instead of efficiency only [106], ‘degrowth’ [107], ‘restraining the present’ [90], ‘maximising persistence’ 108], or a ‘steady-state economy’ [109], which all provide practical examples that—if implemented—could potentially go beyond current destructive human niche construction processes which are solely oriented towards a constant repelling of self-limiting feedbacks from the environment. Instead, these new approaches engage with ways to move beyond destructive and towards regenerative niche construction in terms of, e.g. an active learning to live and flourish with limits (compare, e.g. the doughnut economics in [110]), focusing on what is necessary for a ‘good life for all’ [111] and what not, and better anticipating future detrimental effects from actions set today. In other words, human-designed cultural negative feedback—instead of environmental feedback—which ensures that the system does not expand uncontrollably.

To sum up, life is inherently expansionist, but naturally also confronted with limitations and barriers, like abiotic environmental conditions or competitors, that curb uncontrolled expansion and growth. Every living organism is surrounded by other organisms that provide balancing feedback. However, as we have argued in this article, social-ecological niche construction possesses the ability to ‘outgrow’ and extend many natural barriers and limitations. This ability initially yields positive results for human flourishing, but long-term perspectives are not well understood and potentially catastrophic. In a similar vein, environmental scientist Donella Meadows [112] wrote about unlimited and uncontrolled growth: ‘It's the goal of a cancer cell too. Actually it's the goal of every living population, and only a bad one when it isn't balanced by higher-level negative feedback loops that never let an upstart power-driven entity control the world.’ However, those ‘negative feedback loops’ are suppressed by industrial social-ecological niche construction processes, as described above. In 1972, Meadows and collaborators asserted that: ‘Applying technology to the natural pressures that the environment exerts against any growth process has been so successful in the past that a whole culture has evolved around the principle of fighting against limits rather than learning to live with them’ [113].

To avoid (self-)destructive niche construction processes as an inevitable human predicament and fate, it appears that cultural evolution has to be understood as much more than just focusing on high-tech development, efficiency gains, and the possibility to collaborate globally [79,114]. Keeping in mind the long-term implications of social-ecological inheritance in niche construction, actions and measures set today are to be evaluated against future legacies and lock-ins more thoroughly. The same applies to environmental impacts outsourced to other niches. The heterogeneity, inequality and power imbalances between social-ecological niches that exist simultaneously must be considered explicitly when aiming to steer the totality of the global human niche towards more regenerative processes.

## 5. Conclusion

In this paper, we introduced a conceptual framework for social-ecological niche construction that may support research dealing both about destructive and potentially regenerative processes. First, we argued that social-ecological and human niche construction research can benefit from understanding social-ecological systems as hybrids between the ecological and the sociocultural sphere; further, we showed how established niche construction frameworks can be extended to better accommodate evolutionary processes that allowed human societies to construct niches that repel limiting factors from the environment to a level that endangers future ecological and climatic stabilities on Earth.

We discussed the framework in relation to contemporary sustainability challenges of the Anthropocene. From a social-ecological niche construction perspective, we outlined essential processes for the study of the biophysical aspects of contemporary human niche construction, i.e. an extended spatiotemporal focus beyond on-site human–nature interaction and interactions beyond the biosphere to achieve a more comprehensive picture of how niche construction works in industrialized and highly interconnected societies. Moreover, we discussed where in the industrial social-ecological niche construction process issues of injustice (intra-generational, inter-generational, interspecies) and unsustainability are arising (§2c).

Understanding human–nature interactions and their environmental impact—on different scales—from a biophysical and social-ecological perspective potentially strengthens the analytical capability of niche construction approaches. The combination of niche construction theory from evolutionary biology with insights and methods from social ecology combines crucial aspects of measurable sustainability outcomes with long-term effects and a process orientation. By considering both, it will be possible to better understand how destructive niche construction processes may be overcome while also attempting to establish new and regenerative ones.

## Data Availability

This article has no additional data.

## References

[1] Ellis EC. 2011 Anthropogenic transformation of the terrestrial biosphere. Phil. Trans. R. Soc. A **369**, 1010-1035. (10.1098/rsta.2010.0331) 21282158

[2] Steffen W, Broadgate W, Deutsch L, Gaffney O, Ludwig C. 2015 The trajectory of the Anthropocene: the great acceleration. Anthropocene Rev. **2**, 81-98. (10.1177/2053019614564785)

[3] Steffen W et al. 2018 Trajectories of the Earth system in the Anthropocene. Proc. Natl Acad. Sci. USA **115**, 8252-8259. (10.1073/pnas.1810141115) 30082409 PMC6099852

[4] Crutzen PJ. 2006 The ‘Anthropocene’. In Earth system science in the Anthropocene (eds E Ehlers, T Krafft), pp. 13-18. Berlin, Germany: Springer.

[5] Waters CN et al. 2016 The Anthropocene is functionally and stratigraphically distinct from the Holocene. Science **351**, aad2622. (10.1126/science.aad2622) 26744408

[6] Ellis EC, Magliocca NR, Stevens CJ, Fuller DQ. 2018 Evolving the Anthropocene: linking multi-level selection with long-term social–ecological change. Sustain. Sci. **13**, 119-128. (10.1007/s11625-017-0513-6) 30147774 PMC6086254

[7] Krausmann F, Erb K, Gingrich S, Haberl H, Bondeau A, Gaube V. 2013 Global human appropriation of net primary production doubled in the 20th century. Proc. Natl Acad. Sci. USA **110**, 10 324-10 329. (10.1073/pnas.1211349110) PMC3690849 23733940

[8] Sullivan AP, Bird DW, Perry GH. 2017 Human behaviour as a long-term ecological driver of non-human evolution. Nat. Ecol. Evol. **1**, 1-11. (10.1038/s41559-016-0065) 28812734

[9] Boivin NL, Zeder MA, Fuller DQ, Crowther A, Larson G, Erlandson JM, Denhami T, Petraglia MD. 2016 Ecological consequences of human niche construction: examining long-term anthropogenic shaping of global species distributions. Proc. Natl Acad. Sci. USA **113**, 6388-6396. (10.1073/pnas.1525200113) 27274046 PMC4988612

[10] Kline MA, Waring TM, Salerno J. 2018 Designing cultural multilevel selection research for sustainability science. Sustain. Sci. **13**, 9-19. (10.1007/s11625-017-0509-2) 30147767 PMC6086275

[11] Ellis EC. 2016 Why is human niche construction transforming planet Earth? RCC Perspect. **1**, 63-70.

[12] Snyder BF. 2020 The genetic and cultural evolution of unsustainability. Sustain. Sci. **15**, 1087-1099. (10.1007/s11625-020-00803-z) 32292525 PMC7133775

[13] Xu C, Kohler TA, Lenton TM, Svenning J, Scheffer M. 2020 Future of the human climate niche. Proc. Natl Acad. Sci. USA **117**, 11 350-11 355. (10.1073/pnas.1910114117) PMC7260949 32366654

[14] Fuentes A. 2015 Integrative anthropology and the human niche: toward a contemporary approach to human evolution. Am. Anthropol. **117**, 302-315. (10.1111/aman.12248)

[15] Kendal J, Tehrani JJ, Odling-Smee J. 2011 Human niche construction in interdisciplinary focus. Phil. Trans. R. Soc. B **366**, 785-792. (10.1098/rstb.2010.0306) 21320894 PMC3048995

[16] O'Brien MJ, Bentley RA. 2021 Genes, culture, and the human niche: an overview. Evol. Anthropol. **30**, 40-49. (10.1002/evan.21865) 32986264

[17] Odling-Smee FJ. 1988 Niche-constructing phenotypes. In The role of behavior in evolution (ed. HC Plotkin), pp. 73-132. Cambridge, MA: MIT Press.

[18] Odling-Smee FJ, Laland KN, Feldman MW. 2013 Niche construction: the neglected process in evolution. Princeton, NJ: Princeton University Press.

[19] Laland KN, Odling-Smee J, Myles S. 2010 How culture shaped the human genome: bringing genetics and the human sciences together. Nat. Rev. Genet. **11**, 137-148. (10.1038/nrg2734) 20084086

[20] Perreault C. 2012 The pace of cultural evolution. PLoS ONE **7**, e45150. (10.1371/journal.pone.0045150) 23024804 PMC3443207

[21] Sieferle RP. 2011 Cultural evolution and social metabolism. Geogr. Ann. B **93**, 315-324. (10.1111/j.1468-0467.2011.00385.x)

[22] Richerson PJ, Boyd R. 2017 Cultural inheritance and evolutionary ecology. In Evolutionary ecology and human behavior (eds EA Smith, B Winterhalder), pp. 61-92. New York, NY: Routledge.

[23] Low FM, Gluckman PD, Hanson MA. 2019 Niche modification, human cultural evolution and the anthropocene. Trends Ecol. Evol. **34**, 883-885. (10.1016/j.tree.2019.07.005) 31422891

[24] Steffen W, Crutzen PJ, Mcneill JR. 2007 The Anthropocene: are humans now overwhelming the great forces of nature? AMBIO **36**, 614-621. (10.1579/0044-7447(2007)36[614:taahno]2.0.co;2) 18240674

[25] Hannah M. 2021 Surviving the Anthropocene. In Extinctions: living and dying in the margin of error (ed. M Hannah), pp. 193-210. Cambridge, UK: Cambridge University Press

[26] Boivin N, Crowther A. 2021 Mobilizing the past to shape a better Anthropocene. Nat. Ecol. Evol. **5**, 273-284. (10.1038/s41559-020-01361-4) 33462488

[27] Dorninger C, Abson DJ, Fischer J, von Wehrden H. 2017 Assessing sustainable biophysical human–nature connectedness at regional scales. Environ. Res. Lett. **12**, 055001. (10.1088/1748-9326/aa68a5)

[28] Dorninger C, von Wehrden H, Krausmann F, Bruckner M, Feng K, Hubacek K, Erb K, Abson DJ. 2021 The effect of industrialization and globalization on domestic land-use: a global resource footprint perspective. Glob. Environ. Change **69**, 102311. (10.1016/j.gloenvcha.2021.102311)

[29] Steffen W et al. 2015 Planetary boundaries: guiding human development on a changing planet. Science **347**, 1259855. (10.1126/science.1259855) 25592418

[30] Rockström J, Steffen W, Noone K, Persson A. 2009 A safe operating space for humanity Identifying. Nature **461**, 472-475. (10.1038/461472a) 19779433

[31] Laland KN, Odling-Smee J, Feldman M. 2006 Niche construction, biological evolution and cultural change. Behav. Brain Sci. **23**, 131-146. (10.1017/S0140525X00002417) 11303338

[32] Kendal JR, Tehrani JJ, Odling-Smee J. 2011 Human niche construction. Phil. Trans. R. Soc. Lond. B **366**, 785-792. (10.1098/rstb.2010.0306) 21320894 PMC3048995

[33] Ellis EC. 2015 Ecology in an anthropogenic biosphere. Ecol Monogr **85**, 287-331. (10.1890/14-2274.1)

[34] Pontzer H. 2021 Hotter and sicker: external energy expenditure and the tangled evolutionary roots of anthropogenic climate change and chronic disease. Am. J. Hum. Biol. **33**, e23579. (10.1002/ajhb.23579) 33629785

[35] Meneganzin A, Pievani T, Caserini S. 2020 Anthropogenic climate change as a monumental niche construction process: background and philosophical aspects. Biol. Phil. **35**, 1-20. (10.1007/s10539-020-09754-2)

[36] Jørgensen S. 2023 Evolutionary traps for humanity in the Anthropocene and the pursuit of global sustainability. Phil. Trans. R. Soc. B **378**, 20220261. (10.1098/rstb.2022.0261)

[37] Haberl H, Fischer-Kowalski M, Krausmann F, Winiwarter V. 2016 Social ecology: society-nature relations across time and space. Berlin, Germany: Springer International Publishing.

[38] Preiser R, Biggs R, De Vos A, Folke C. 2018 Social-ecological systems as complex adaptive systems: organizing principles for advancing research methods and approaches. Ecol. Soc. **23**, 4. (10.5751/ES-10558-230446)

[39] Hornborg A. 2001 The power of the machine: global inequalities of economy, technology, and environment. Walnut Creek, CA: Rowman Altamira.

[40] Hornborg A. 2015 The political ecology of the Technocene: uncovering ecologically unequal exchange in the world-system. In The Anthropocene and the global environmental crisis (eds C Hamilton, F Gemenne, C Bonneuil), pp. 57-69. Abingdon, UK: Routledge.

[41] Krausmann F, Lauk C, Haas W, Wiedenhofer D. 2018 From resource extraction to outflows of wastes and emissions: the socioeconomic metabolism of the global economy, 1900–2015. Global Environ. Change **52**, 131-140. (10.1016/j.gloenvcha.2018.07.003) PMC6333294 30679887

[42] Schandl H et al. 2018 Global material flows and resource productivity: forty years of evidence. J. Indust. Ecol. **22**, 827-838. (10.1111/jiec.12626)

[43] Odling-Smee FJ, Laland KN, Feldman MW. 2003 Niche construction: the neglected process in evolution. Monographs in population biology 37th edn. Princeton, NJ: Princeton University Press.

[44] Laland KN, Boogert N, Evans C. 2014 Niche construction, innovation and complexity. Environ. Innov. Soc. Transit. **11**, 71-86. (10.1016/j.eist.2013.08.003)

[45] Laland KN, O'Brien MJ. 2011 Cultural niche construction: an introduction. Biol. Theory **6**, 191-202. (10.1007/s13752-012-0026-6)

[46] Fischer-Kowalski M, Weisz H. 1999 Society as hybrid between material and symbolic realms. Toward a theoretical framework of society-nature interaction. Adv. Hum. Ecol. **8**, 215-251.

[47] Fischer-Kowalski M, Krausmann F, Pallua I. 2014 A sociometabolic reading of the Anthropocene: modes of subsistence, population size and human impact on Earth. Anthropocene Rev. **1**, 8-33. (10.1177/2053019613518033)

[48] Pinton R, Varanini Z, Nannipieri P. 2007 The rhizosphere: biochemistry and organic substances at the soil-plant interface. Boca Raton, CA: CRC Press.

[49] Gerbault P, Liebert A, Itan Y, Powell A, Currat M, Burger J, Swallow DM, Thomas MG. 2011 Evolution of lactase persistence: an example of human niche construction. Phil. Trans. R. Soc. B **366**, 863-877. (10.1098/rstb.2010.0268) 21320900 PMC3048992

[50] Fischer-Kowalski M, Haberl H. 1998 Sustainable development: socio-economic metabolism and colonization of nature. Int. Soc. Sci. J. **50**, 573-587. (10.1111/1468-2451.00169)

[51] Fischer-Kowalski M, Haberl H. 1993 Metabolism and colonization. Modes of production and the physical exchange between societies and nature. Innovation **6**, 415-442. (10.1080/13511610.1993.9968370)

[52] Odling-Smee J, Laland KN. 2011 Ecological inheritance and cultural inheritance: what are they and how do they differ? Biol. Theory **6**, 220-230. (10.1007/s13752-012-0030-x)

[53] Laland K, Odling-Smee J, Endler J. 2017 Niche construction, sources of selection and trait coevolution. Interface Focus **7**, 20160147. (10.1098/rsfs.2016.0147) 28839920 PMC5566808

[54] Waring TM, Kline MA, Goff S, Gowdy J, Jacquet J. 2015 A multilevel evolutionary framework for sustainability analysis. Ecol. Soc. **20**, 34. (10.5751/ES-07634-200234)

[55] Gowdy J, Krall L. 2016 The economic origins of ultrasociality. Behav. Brain Sci. **39**, 1-60. (10.1017/S0140525X1500059X) 25915060

[56] Krausmann F. 2004 Milk, manure, and muscle power: livestock and the transformation of preindustrial agriculture in Central Europe. Hum. Ecol. **32**, 735-772. (10.1007/s10745-004-6834-y)

[57] Hornborg A. 2016 Global magic: technologies of appropriation from ancient Rome to Wall Street. New York, NY: Palgrave Macmillan.

[58] Krausmann F, Langthaler E. 2019 Food regimes and their trade links: a socio-ecological perspective. Ecol. Econ. **160**, 87-95. (10.1016/j.ecolecon.2019.02.011)

[59] Brolin J, Kander A. 2020 Global trade in the Anthropocene: a review of trends and direction of environmental factor flows during the Great Acceleration. Anthropocene Rev. **9**, 71-110. (10.1177/2053019620973711)

[60] Gatti RC et al. 2021 Diversity lost: COVID-19 as a phenomenon of the total environment. Sci. Total Environ. **756**, 144014. (10.1016/j.scitotenv.2020.144014) 33279199

[61] Brooks DR, Hoberg EP, Boeger WA. 2019 The Stockholm paradigm. Chicago, IL: University of Chicago Press.

[62] Keys PW, Galaz V, Dyer M, Matthews N, Folke C, Nyström M, Cornell SE. 2019 Anthropocene risk. Nat. Sustain. **2**, 667-673. (10.1038/s41893-019-0327-x)

[63] Fischer-Kowalski M, Haberl H. 2007 Socioecological transitions and global change: trajectories of social metabolism and land use. Cheltenham, UK: Edward Elgar Publishing.

[64] Fischer-Kowalski M. 2011 Analyzing sustainability transitions as a shift between socio-metabolic regimes. Environ. Innov. Soc. Transit. **1**, 152-159. (10.1016/j.eist.2011.04.004) 27066392 PMC4802507

[65] Elhacham E, Ben-Uri L, Grozovski J, Bar-On YM, Milo R. 2020 Global human-made mass exceeds all living biomass. Nature **588**, 442-444. (10.1038/s41586-020-3010-5) 33299177

[66] Krausmann F, Wiedenhofer D, Lauk C, Haas W, Tanikawa H, Fishman T, Miatto A, Schandl H, Haberl H. 2017 Global socioeconomic material stocks rise 23-fold over the 20th century and require half of annual resource use. Proc. Natl Acad. Sci. USA **114**, 1880-1885. (10.1073/pnas.1613773114) 28167761 PMC5338421

[67] Liu J et al. 2013 Framing sustainability in a telecoupled world. Ecol. Soc. **18**, 26. (10.5751/ES-05873-180226)

[68] Dorninger C, Hornborg A, Abson DJ, von Wehrden H, Schaffartzik A, Giljum S, Engler J, Feller RL, Hubacek K. 2021 Global patterns of ecologically unequal exchange: implications for sustainability in the 21st century. Ecol. Econ. **179**, 106824. (10.1016/j.ecolecon.2020.106824)

[69] Cumming GS, von Cramon-Taubadel S. 2018 Linking economic growth pathways and environmental sustainability by understanding development as alternate social–ecological regimes. Proc. Natl Acad. Sci. USA **115**, 9533-9538. (10.1073/pnas.1807026115) 30185564 PMC6156676

[70] Laland K, Matthews B, Feldman MW. 2016 An introduction to niche construction theory. Evol. Ecol. **30**, 191-202. (10.1007/s10682-016-9821-z) 27429507 PMC4922671

[71] Cumming GS, Buerkert A, Hoffmann EM, Schlecht E, von Cramon-Taubadel S, Tscharntke T. 2014 Implications of agricultural transitions and urbanization for ecosystem services. Nature **515**, 50-57. (10.1038/nature13945) 25373674

[72] Ilankoon I, Ghorbani Y, Chong MN, Herath G, Moyo T, Petersen J. 2018 E-waste in the international context—a review of trade flows, regulations, hazards, waste management strategies and technologies for value recovery. Waste Manag. **82**, 258-275. (10.1016/j.wasman.2018.10.018) 30509588

[73] Martinez-Alier J. 2001 Mining conflicts, environmental justice, and valuation. J. Hazard. Mater. **86**, 153-170. (10.1016/S0304-3894(01)00252-7) 11532364

[74] Friis C, Nielsen JØ, Otero I, Haberl H, Niewöhner J, Hostert P. 2016 From teleconnection to telecoupling: taking stock of an emerging framework in land system science. J. Land Use Sci. **11**, 131-153. (10.1080/1747423X.2015.1096423)

[75] Krausmann F, Schandl H, Eisenmenger N, Giljum S, Jackson T. 2017 Material flow accounting: measuring global material use for sustainable development. Annu. Rev. Environ. Resour. **42**, 647-675. (10.1146/annurev-environ-102016-060726)

[76] Haberl H, Erb K, Krausmann F. 2014 Human appropriation of net primary production: patterns, trends, and planetary boundaries. Annu. Rev. Environ. Resour. **39**, 363-391. (10.1146/annurev-environ-121912-094620)

[77] Hubacek K, Feng K, Minx JC, Pfister S, Zhou N. 2014 Teleconnecting consumption to environmental impacts at multiple spatial scales: research frontiers in environmental footprinting. J. Indust. Ecol. **18**, 7-9. (10.1111/jiec.12082)

[78] Prell C, Sun L, Feng K, He J, Hubacek K. 2017 Uncovering the spatially distant feedback loops of global trade: a network and input-output approach. Sci. Total Environ. **586**, 401-408. (10.1016/j.scitotenv.2016.11.202) 28233613

[79] Ellis EC. 2023 The Anthropocene condition: evolving through social-ecological transformations. Phil. Trans. R. Soc. B **378**, 20220255. (10.1098/rstb.2022.0255) PMC10645118 37952626

[80] Ehrlich PR. 2009 Cultural evolution and the human predicament. Trends Ecol. Evol. **24**, 409-412. (10.1016/j.tree.2009.03.015) 19577322

[81] Tsing AL. 2015 The mushroom at the end of the world. On the possibility of life in capitalist ruins. Princeton, NJ: Princeton University Press.

[82] Bélanger J, Pilling D, (eds). 2019 The state of the world's biodiversity for food and agriculture. Rome, Italy: FAO Commission on Genetic Resources for Food and Agriculture Assessments.

[83] Hickel J, Dorninger C, Wieland H, Suwandi I. 2022 Imperialist appropriation in the world economy: drain from the global south through unequal exchange, 1990–2015. Global Environ. Change **73**, 102467. (10.1016/j.gloenvcha.2022.102467)

[84] Höök M, Tang X. 2013 Depletion of fossil fuels and anthropogenic climate change—a review. Energy Pol. **52**, 797-809. (10.1016/j.enpol.2012.10.046)

[85] Giljum S, Eisenmenger N. 2004 North-South trade and the distribution of environmental goods and burdens: a biophysical perspective. J. Environ. Dev. **13**, 73-100. (10.1177/1070496503260974)

[86] Dorninger C, Hornborg A. 2015 Can EEMRIO analyses establish the occurrence of ecologically unequal exchange? Ecol. Econ. **119**, 414-418. (10.1016/j.ecolecon.2015.08.009)

[87] Sieferle RP. 2006 Die Risikospirale. Wissenschaft Umwelt **10**, 157-174.

[88] Winiwarter V, Schmid M, Haberl H, Singh SJ. 2016 Why legacies matter: merits of a long-term perspective. In Social ecology (eds H Haberl, M Fischer-Kowalski, F Krausmann, V Winiwarter), pp. 149-168. Berlin, Germany: Springer.

[89] Levin K, Cashore B, Bernstein S, Auld G. 2012 Overcoming the tragedy of super wicked problems: constraining our future selves to ameliorate global climate change. Pol. Sci. **45**, 123-152. (10.1007/s11077-012-9151-0)

[90] Lazarus RJ. 2008 Super wicked problems and climate change: restraining the present to liberate the future. Cornell L. Rev. **94**, 1153-1234.

[91] Mitteroecker P, Huttegger SM, Fischer B, Pavlicev M. 2016 Cliff-edge model of obstetric selection in humans. Proc. Natl Acad. Sci. USA **113**, 14 680-14 685. (10.1073/pnas.1612410113) 27930310 PMC5187675

[92] Harari YN. 2014 Sapiens: a brief history of humankind. New York, NY: Random House.

[93] Jiborn M, Kander A, Kulionis V, Nielsen H, Moran DD. 2018 Decoupling or delusion? Measuring emissions displacement in foreign trade. Global Environ. Change **49**, 27-34. (10.1016/j.gloenvcha.2017.12.006)

[94] Haberl H et al. 2020 A systematic review of the evidence on decoupling of GDP, resource use and GHG emissions, part II: synthesizing the insights. Environ. Res. Lett. **15**, 065003. (10.1088/1748-9326/ab842a)

[95] Kaaronen RO, Mulder MB, Waring T. 2022 Applying cultural evolution to address climate and environmental challenges. Preprint. (10.31219/osf.io/u7hvj)

[96] Fletcher R, Rammelt C. 2017 Decoupling: a key fantasy of the post-2015 sustainable development agenda. Globalizations **14**, 450-467. (10.1080/14747731.2016.1263077)

[97] Boogert NJ, Paterson DM, Laland KN. 2006 The implications of niche construction and ecosystem engineering for conservation biology. Bioscience **56**, 570-578. (10.1641/0006-3568(2006)56[570:TIONCA]2.0.CO;2)

[98] Catton WR, Dunlap RE. 1978 Environmental sociology: a new paradigm. Am. Sociol. **13**, 41-49.

[99] Haberl H, Fischer-Kowalski M, Krausmann F, Martinez-Alier J, Winiwarter V. 2011 A socio-metabolic transition towards sustainability? Challenges for another Great Transformation. Sustain. Dev. **19**, 1-14. (10.1002/sd.410)

[100] Harari YN. 2016 Homo deus: a brief history of tomorrow. New York, NY: Random House.

[101] Maienschein J, MacCord K. 2022 What Is regeneration?. Chicago, IL: University of Chicago Press.

[102] Carlson BM. 2011 Principles of regenerative biology. San Diego, CA: Elsevier.

[103] Bennett EM, Biggs R, Peterson GD, Gordon LJ. 2021 Patchwork Earth: navigating pathways to just, thriving, and sustainable futures. One Earth **4**, 172-176. (10.1016/j.oneear.2021.01.004)

[104] Bennett EM et al. 2016 Bright spots: seeds of a good Anthropocene. Front. Ecol. Environ. **14**, 441-448. (10.1002/fee.1309)

[105] Brand U et al. 2021 From planetary to societal boundaries: an argument for collectively defined self-limitation. Sustainability: Sci. Pract. Pol. **17**, 264-291. (10.1080/15487733.2021.1940754)

[106] Sandberg M. 2021 Sufficiency transitions: a review of consumption changes for environmental sustainability. J. Clean Prod. **293**, 126097. (10.1016/j.jclepro.2021.126097)

[107] D'Alisa G, Demaria F, Kallis G. 2014 Degrowth: a vocabulary for a new era. New York, NY: Routledge.

[108] Lenton T, Scheffer M. 2023 Spread of the cycles: a feedback perspective on the Anthropocene. Phil. Trans. R. Soc. B **378**, 20220254. (10.1098/rstb.2022.0254) PMC10645129 37952624

[109] Daly HE (ed.) 1973 Toward a steady-state economy. San Francisco, CA: WH Freeman.

[110] Raworth K. 2017 A doughnut for the Anthropocene: humanity's compass in the 21st century. Lancet Planet Health **1**, e48-e49. (10.1016/S2542-5196(17)30028-1) 29851576

[111] Neill DWO, Fanning AL, Lamb WF, Steinberger JK. 2018 A good life for all within planetary boundaries. Nat. Sustain. **1**, 88-95. (10.1038/s41893-018-0021-4)

[112] Meadows D. 1999 Leverage points. Places to intervene in a system. Hartland, VT: The Sustainability Institute.

[113] Meadows DH, Meadows DH, Randers J, Behrens III WW. 1972 The limits to growth: a report to the Club of Rome's project on the predicament of mankind. New York, NY: Universe Books.

[114] Waring T, Wood Z, Szathmary E. 2023 Characteristic processes of human evolution may have caused the Anthropocene and obstruct its collective global solutions. Phil. Trans. R. Soc. B **378**, 20220259. (10.1098/rstb.2022.0259) PMC10645123 37952628

